# Blockage of Extracellular Signal-Regulated Kinase Exerts an Antitumor Effect via Regulating Energy Metabolism and Enhances the Efficacy of Autophagy Inhibitors by Regulating Transcription Factor EB Nuclear Translocation in Osteosarcoma

**DOI:** 10.3389/fcell.2021.650846

**Published:** 2021-08-03

**Authors:** Man Zhang, Yang Bai, Chang Xu, Yiying Qi, Jiahong Meng, Wenkan Zhang, Hang Su, Weiqi Yan

**Affiliations:** ^1^Department of Orthopedic Surgery, The Second Affiliated Hospital, Zhejiang University School of Medicine, Hangzhou, China; ^2^Orthopedics Research Institute of Zhejiang University, Hangzhou, China; ^3^Key Laboratory of Motor System Disease Research and Precision Therapy of Zhejiang Province, Hangzhou, China; ^4^The Second Affiliated Hospital, School of Medicine, Zhejiang University, Hangzhou, China

**Keywords:** ERK, transcription factor EB, autophagy, osteosarcoma, energy metabolism

## Abstract

Accumulating evidence suggests that extracellular signal-regulated kinase (ERK) is a valuable target molecule for cancer. However, antitumor drugs targeting ERK are still in their clinical phase and no FDA-approved medications exist. In this study, we identified an ERK inhibitor (ERKi; Vx-11e) with potential antitumor activities, which was reflected by the inhibition in the survival and proliferation of Osteosarcoma (OS) cells. Mechanistically, the ERKi regulated autophagic flux by promoting the translocation of transcription factor EB (TFEB) in OS cells, thereby increasing the dependence of OS cells on autophagy and sensitivity to treatment with autophagy inhibitors in OS. Besides, we also found that the ERKi could regulate mitochondrial apoptosis through the ROS/mitochondria pathway and aerobic glycolysis in OS, which also increases the dependence of OS cells on autophagy to clear metabolites to a certain extent. These results may provide a reference for the clinically improved efficacy of ERKis in combination with autophagy inhibitors in the treatment of OS and indicate its potential as a therapeutic agent.

## Introduction

Osteosarcoma (OS) is a highly malignant bone tumor characterized by early metastasis. The incidence of OS is rising rapidly, and it is known to have the highest mortality rate among all cancer types, especially in children and adolescents (Zhang, 2019). With the development of several therapies such as surgery, chemotherapy, radiation, targeted therapy, and immunotherapy, the survival rate of OS patients has increased; however, this rate still is unsatisfactory (Ritter and Bielack, 2010). There are several problems that need to be overcome among the current treatment strategies, such as effective inhibition of the metastasis, and the reduction in severe side effects and drug resistance caused by single-drug chemotherapy. Therefore, it is necessary to explore new treatment methods and strategies to treat OS. Particularly, therapies focusing on combination therapy are urgently needed.

As an important cellular recycling mechanism, autophagy is responsible for the degradation of unnecessary or dysfunctional proteins and organelles within cells (Glick et al., 2010). Autophagy promotes the growth and survival of various cancer cells as it increases their ability to support cellular metabolism. Studies have shown that activation of autophagy is beneficial for cellular growth and survival in breast cancer (Wei et al., 2011), glioblastoma (Jawhari et al., 2016), and hematological malignancies (Karvela et al., 2016) by the maintenance of glycolytic capacity. Autophagy restricts oxidative stress, prevents intra-tumoral necrosis and local inflammation in response to stress, and regulates glycolysis to promote tumor growth (Mowers et al., 2018). However, in OS, autophagy acts as both a pro-tumoral and antitumoral process. A previous study suggests that autophagy facilitates glycolytic metabolism and increases lung colonization by OS cells *via* upregulating the expression of intercellular adhesion molecule-1 (ICAM-1) and enhancing tumor metastasis (Itoh et al., 2018). Another study has shown that autophagy induces cell apoptosis in OS (Meschini et al., 2008). Based on these studies, the role of autophagy in the growth of OS cells poses several challenges that need to be further explored. Transcription factor EB (TFEB) is a subfamily of the MiT/TFE basic helix–loop–helix leucine-zipper (bHLH-Zip) family of transcription activators (Sardiello, 2016). TFEB regulates various cellular processes, including autophagy (Martini-Stoica et al., 2016), lysosomal biogenesis (Chao et al., 2018), and energy metabolism (Pastore et al., 2017). TFEB activity is tightly controlled through TFEB nuclear translocation. Multiple environmental stimuli, including proinflammatory agents, mitochondrial and oxidative stress, can promote TFEB nuclear translocation to increase its transcription activity. However, its mechanistic interaction with autophagy in OS remains elusive.

Mitochondria are the primary sites of energy metabolism in eukaryotic cells that provide energy for cell growth, proliferation, and other biological activities (Horbay and Bilyy, 2016). However, given that the “Warburg effect” is one of the basic characteristics of tumor cells, the activity in mitochondria is also necessary for many tumor cells (Hsu et al., 2016). Additionally, mitochondria control calcium homeostasis and redox reactions and participate in transcriptional regulation (Hsu et al., 2016; Porporato et al., 2018). Moreover, a dysregulated mitochondrial respiratory chain or exposure to certain chemicals affects the mitochondrial DNA in cancer cells, thereby inducing mitochondrial dysfunction and promoting cancer progression to a chemoresistant or invasive phenotype (Giang et al., 2013; Hsu et al., 2016). Therefore, mitochondria are a potential target for the development of new anticancer drugs. Mitochondria exist in dynamic equilibrium by continuous fusion and fission events. During this process, mitochondrial morphology changes, resulting in the elimination of damaged mitochondria and the production of new ones (Horbay and Bilyy, 2016). Studies have shown that the induction of mitochondrial dysfunction in OS cells is associated with the modulation of mitochondrial fission/fusion proteins that exert anticancer effects (Huang et al., 2014). However, another study shows that the suppression of mitochondrial function contributes to the Warburg effect in OS cells (Giang et al., 2013; Gorska-Ponikowska et al., 2018). Hence, the role of mitochondria in OS remains slightly controversial and needs to be further explored. Mitogen-activated protein kinases (MAPKs) regulate the growth, proliferation, and apoptosis of cells (Cuadrado and Nebreda, 2010; Santos and Crespo, 2018), and the excessive activation of MAPKs is found in several tumors (Wagner and Nebreda, 2009; Yong et al., 2009). Extracellular signal-regulated kinase (ERK)1/2 is one of the main members of the MAPK family (Kim and Choi, 2010). The expression level of ERK is increased in various human tumors, and interferences with the ERK pathway may be a potential therapeutic strategy for treating these cancers (Samatar and Poulikakos, 2014; Santos and Crespo, 2018). Activation of the ERK pathway has been confirmed to promote autophagy in cancer cells, and some literature suggests that the inhibition of ERK inhibits aerobic glycolysis and induces stress, leading to the death of cancer cells (Marchetti et al., 2018; Aloia et al., 2019). However, similar findings have rarely been reported in OS. Therefore, whether ERK has an effect on the metabolism in OS is not fully understood and therefore warrants the elucidation of its underlying mechanism.

## Materials and Methods

### Cell Culture and siRNA Transfection

The human OS cell lines (HOSs), U2-OS (U2), 143B, and MG-63, and the murine spontaneous OS cell line K7M2 were obtained from the Cell Bank of the Chinese Academy of Sciences (Shanghai, China^1^) and maintained at 37°C and 5% CO_2_ in a humid environment. The cells were cultured in Dulbecco’s modified Eagle’s medium (DMEM), supplemented with 10% fetal bovine serum and antibiotics (1% penicillin/streptomycin). TFEBsiRNA was delivered into the OS cell line using Lipofectamine 2000. TFEB siRNA was constructed by TSINGKE (Beijing, China). The sequences were as follows: TFEBsiRNA1, F:GAUGUCAUUGACAACAUUATT; R:UAAUGUUGUCAAUGACAUCTT and TFEBsiRNA2, F:CC AAGAAGGAUCUGGACCUTT; R:AGGUCCAGAUCCUUCU UGGTT. Vx-11e (APExBiO) was dissolved in dimethyl sulfoxide (DMSO), and the NC group (DMSO) was treated with the same amount of DMSO. Hydroxychloroquine (HCQ) was purchased from Aladdin. LysoTracker was obtained from Solarbio. Lactic Acid assay kit and Glucose Uptake assay kit were obtained from Nanjing JianCheng Bioengineering Institute.

### Cell Proliferation and Cytotoxicity Assays

Cells were seeded into a 96-well plate and treated with different drugs. After 24 h of coculture, the Cell Counting Kit-8 (Dojindo, Japan) dye was added. For colony formation, cells were seeded into a six-well plate at a density of 500–1,000 cells/well. The plate was then shaken well to maintain sufficient distance between individual cells and incubated under different treatment conditions for 14–21 days. The colonies were visualized under a microscope and photographed.

### Cell Invasion and Migration Assays

The wound-healing assay was used to detect cell migration. A wound was made using a pipette tip when the cell proliferation reached approximately 80% confluence in the six-well plate. The cells were then visualized and photographed. Transwell chambers (Corning Life Science, United States) were used to determine cell invasion and migration abilities.

### Determination of Glutathione Peroxidase (GPx), Glutathione (GSH), and Reactive Oxygen Species (ROS)

Oxidative conversion of 2′,7′-dichlorodihydrofluorescein diacetate (DCFH-DA) (Beyotime Biotech) was used to investigate the levels of intracellular ROS. The detailed procedure was performed as per the manufacturer’s instructions. Briefly, the cells were plated in a 6-well plate and subjected to different drug treatments for 6 h and 24 h and then incubated with DCFH-DA for 15 min. Next, the cells from each well were detected using fluorescence microplate assay. Samples were harvested for the measurement of GPx and GSH content spectrophotometrically using the respective assay kits (Beyotime Biotech Inc., Jiangsu, China).

### Mitochondrial Transmembrane Potential Assay

To detect the loss of mitochondrial transmembrane potential (ΔΨm), the fluorescent probe, JC-1 (Beyotime, Jiangsu, China), was used. Briefly, the cells were seeded in six-well plates, stained with JC-1 staining solution for 15 min at 37°C, and washed with phosphate-buffered saline (PBS). Thereafter, images were obtained using fluorescence microscopy (Olympus).

### Transmission Electron Microscopy (TEM) Analysis

Cells were collected and fixed in 2.5% glutaraldehyde for 24 h at 4°C, and the intracellular and morphological changes in the subcellular ultrastructure were investigated by TEM. Next, the cells were again fixed with 1% osmium tetroxide for 2 h at room temperature and then dehydrated in different concentrations and gradients of alcohol. The sample was then finally embedded in Spurr’s resin to form pellets. Ultrastructural analysis was performed using TEM (Hitachi H-7650, Japan).

### Flow Cytometry

Flow cytometry was performed as per the manufacturer’s protocols. Thereafter, the cells were harvested and stained with Annexin V conjugated with APC and 7-aminoactinomycin D (Multi-Sciences, China). Next, FACScan flow cytometry (Becton-Dickinson, United States) was used to analyze the samples and measure the percentage of apoptotic cells.

### Glycolysis Measurement

Glucose Uptake Assay Kit and Lactic Acid Assay Kit were used to measure glucose utilization and glycolysis, respectively. Mitochondrial respiration was tested by measuring the oxygen consumption rate (OCR) in OS cells. In brief, cells were added to a 2-mL chamber (3–4 × 10^6^ cells/chamber). The oxygen flux (pmol O_2_/min) of cells is proportional to oxygen consumption, which was recorded continuously using oxygraphy (Oroboros Oxygraph-2k). Data were analyzed using one-way ANOVA and Student’s *t*-test in DatLab software 7.

### *In vivo* Experiments

All animal experiments complied with the guidelines and followed a protocol approved by the Research Ethics Committee of Zhejiang University, China. Nude mice (4-weeks-old, male, Shanghai Laboratory Animal Center, Chinese Academy of Sciences, Shanghai, China) were raised under specific pathogen-free conditions. The mice were inoculated with 2 × 10^6^ K7M2 cells via the marrow cavity of the right tibia. After 7 days, these mice were randomized into three groups. The mice received daily ERKi (50 mg/kg, p.o) and HCQ (60 mg/kg, p.o). Tibial tumors were harvested 2 weeks after treatment, and each tumor was weighed. The tumor sizes were calculated as volume (cm^3^) = [π × width^2^ (cm^2^) × length (cm)]/6.

### Western Blotting and Co-immunoprecipitation (IP)

Cellular proteins (60 μg) were separated using 10% SDS-PAGE and transferred onto a polyvinylidene fluoride membrane (Bio-Rad Laboratories). After blocking (5% fat-free milk) for 1.5 h at room temperature, the membranes were incubated with the following primary antibodies: active-caspase-3 (1:1,000, Cst), Bcl-XL (1:1,000, Cst), C-MYC (1:1,000, Proteintech Group, Inc), Bax (1:500, bioss), Beclin-1 (1:500, Bioss), LC3 (1:1,000, Proteintech Group, Inc), ERK (1:1,000, Cst), p-ERK (1:1,000, Cst), and GAPDH (Cat: RT1210-1, Huabio) overnight at 4°C. The membranes were then washed with TBST and incubated with a secondary antibody for 1 h at room temperature. The signals were visualized with the ChemiDoc^TM^ XRS + Imaging System (BioRad Laboratories, Hercules, CA, United States), and the densities of the immunoreactive bands were analyzed using ImageJ software (NIH, Bethesda, MD, United States). For IP, the commercial antibody was added to whole-cell lysates (WCLs), followed by co-incubation at 4°C for 12 h. Subsequently, the protein A/G agarose beads were added to the cell lysate and co-incubated at 4°C for 2 h. Western blotting was performed to detect the precipitated and co-precipitated proteins.

### Immunofluorescence

Cells were placed on a coverslip and fixed with 4% paraformaldehyde at room temperature. After blocking with 5% bovine serum albumin (BSA) in PBS for 30 min, the samples were incubated with the following primary antibodies: active-caspase-3 (1:100, CST), active-caspase-9 (1:100, CST), LC3B (1:300, Proteintech Group, Inc), and Mff (1:300, Proteintech Group, Inc) at 4°C overnight. After washing three times with PBS, the samples were incubated with the secondary antibody (1:1,000, Huabio), and the sections were then re-stained with anti-fluorescent quench sealant (Yeasen). The fluorescence images were captured using confocal laser microscopy (Nikon, A1PLUS, Tokyo, Japan).

### Hematoxylin and Eosin (H&E)

Tissues were collected and fixed in 4% (w/v) paraformaldehyde for 24 h and embedded in paraffin wax. Sections were then prepared and mounted on slides for histopathological examination. H&E staining and Cresyl violet staining were performed according to the manufacturer’s instructions. Images were obtained using light microscopy.

### Statistical Analysis

Data are presented as means ± SEM. Differences between two groups were determined using Student’s *t-*test, whereas one-way ANOVA followed by Dunnett’s *post hoc* test was used to determine differences among multiple groups. A *p* < 0.05 was considered significant.

## Results

### ERK Inhibition Elevates Autophagic Flux Through TFEB

**FIGURE 1:**
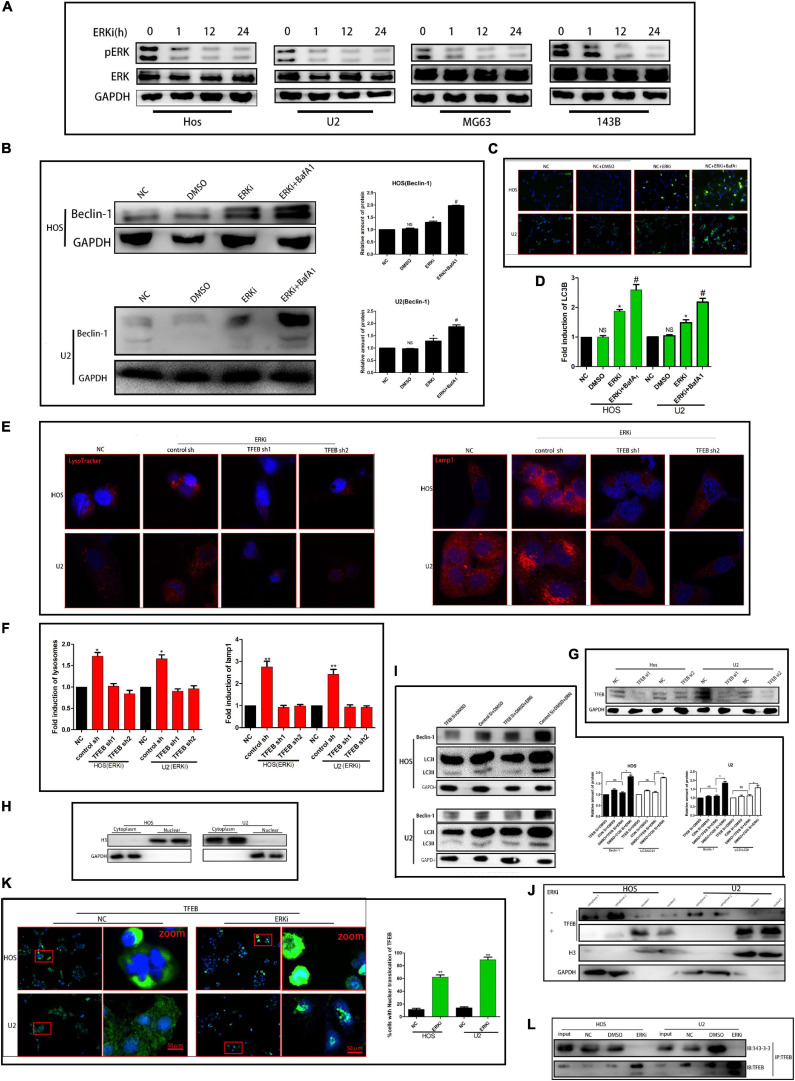
ERKi treatment increases the autophagic flux through TFEB in OS cells. **(A)** Protein expression of p-ERK and ERK after ERKi treatment. **(B)** Protein expression and densitometric quantification of beclin-1 after ERKi and bafilomycin A1 treatment. GAPDH was used as a loading control and for band density normalization. **(C,D)** Immunofluorescence staining of LC3B (green) in OS cells 24 h after ERKi treatment. **(E,F)** Immunofluorescence staining of mitochondria. **(G–I)** Western blotting. **(K)** Immunofluorescence staining of TFEB. **(J)** Immunoblots for TFEB in the cytoplasmic/nuclear fractions of OS cells treated with ERKi. **(L)** ERKi disrupts TFEB interaction with 14–3–3. WCLs of OS cells immunoprecipitated (IP) with anti-TFEB, followed by immunoblotting (IB) with antibodies against 14–3–3 and TFEB (*n* = 5 per group; **p < 0.01 versus NC group; *p < 0.05 versus NC group).

To determine the effect of ERK on the growth of OS cells, we treated OS cells with an ERK-selective inhibitor, Vx-11e (3 μM). As shown in Figure 1A, Vx-11e restrained the phosphorylation of ERK in the OS cell line, resulting in an increase in the conversion of LC3 from LC3-I to LC3-II (Figures 1C,D). Results from western blotting showed that the expression of Beclin-1 protein was increased after Vx-11e treatment (Figure 1B), and the knockdown of TFEB by small interfering RNA reversed the expression of Beclin-1 protein (Figures 1G,I); however, bafilomycin A_1_ (BafA_1_, 100 nM), a lysosomal function inhibitor, increases the expression of the protein (Figure 1B). The lysosomes expand in Vx-11e-treated cells, as indicated by the lysosome-associated membrane protein 1 (Lamp1) and LysoTracker staining, but not in TFEB-knockdown cells (Figures 1E,F). Further, we also found that Vx-11e treatment triggered the nuclear translocation of TFEB (Figures 1H,J,K) and abolished the 14-3-3/TFEB complex formation in OS cells (Figure 1L).

### ERK Inhibition Restrains the Activity of OS Cells

**FIGURE 2:**
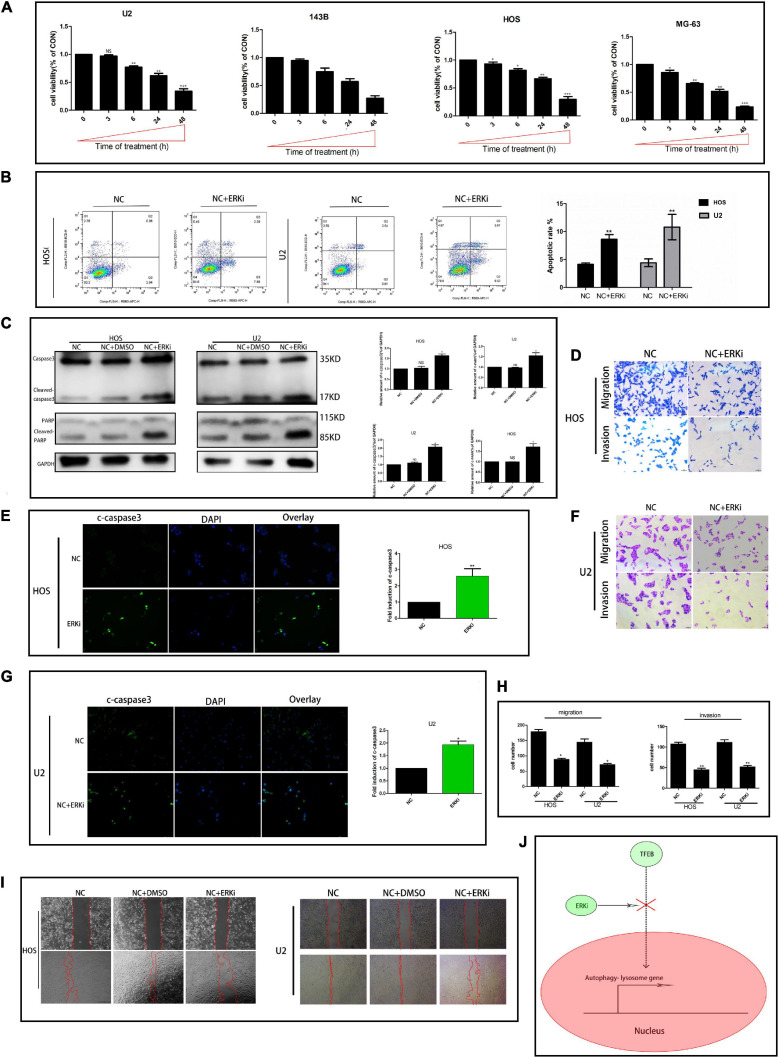
ERK inhibition restrains the activity of OS cells. **(A)** CCK8 proliferation analysis. **(B)** Flow cytometry to determine the total proportion of apoptotic cells in OS. **(C)** Protein expression of caspase3, c-caspase3, c-PARP, and PARP after treatment with ERKi. GAPDH was used as a loading control and for band density normalization. **(E,G)** Immunofluorescence staining of c-caspase3 (green) in OS cells 24 h after ERKi treatment. **(D,F,H)** Migration and invasion. **(I)** Wound-healing assay in different groups. **(J)** Schematic representation of ERKi effect on autophagy in OS cells. Scale bar = 50 μm (*n* = 5 per group; ***p < 0.001 versus NC group; **p < 0.01 versus NC group; *p < 0.05 versus NC group).

The survival rate of OS cells treated with ERKi gradually decreased in a time-dependent manner (Figure 2A). Cellular apoptosis was detected using flow cytometry after annexin V-APC/7-AAD (MultiSciences, China) double staining. The apoptotic abilities of OS cells were significantly higher than those of NC cells (Figure 2B). We obtained similar results for the expression of cleaved caspase-3, cleaved poly-ADP-ribose polymerase (PARP), and PARP protein levels using western blotting and immunofluorescence assay (Figures 2C,E,G). The invasion and migration of the ERKi groups were significantly abrogated in OS cells compared with those in NC cells (Figures 2D,F,H), which was similar to the results observed in the wound-healing assay (Figure 2I).

### ERK Inhibition Impairs Mitochondrial Activity

**FIGURE 3:**
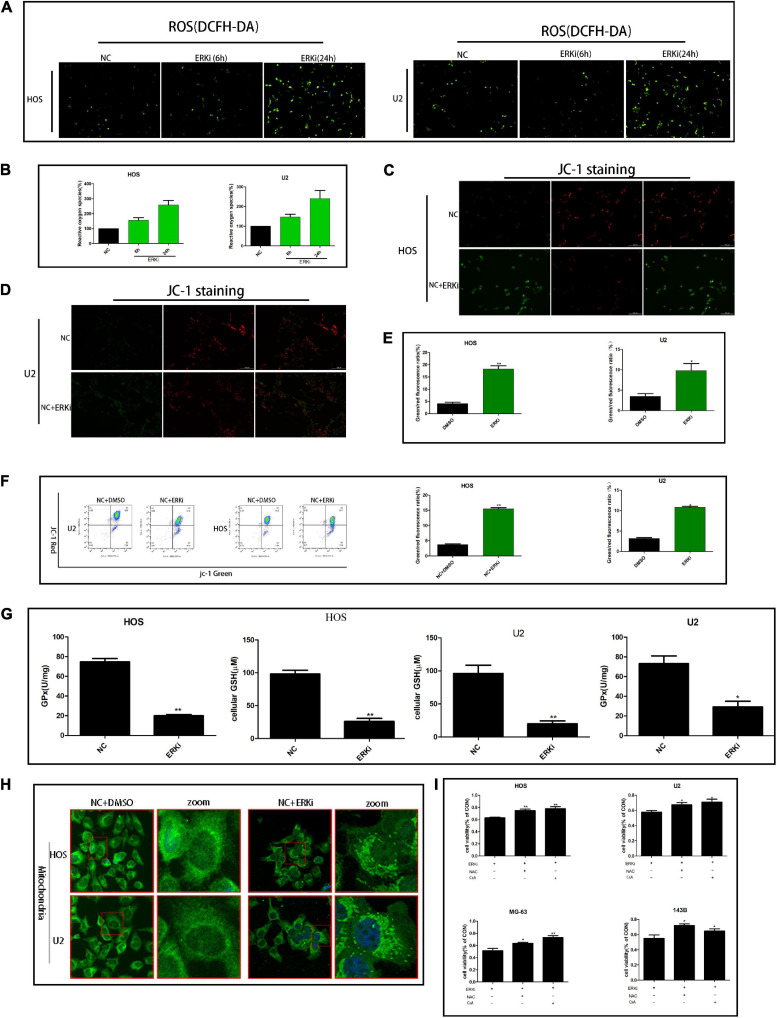
ERK inhibition regulates the mitochondrial activity of OS cells. **(A,B)** ROS fluorescence probe analysis. **(C–E)** JC-1 staining for comparing mitochondrial depolarization of OS cell lines among groups**. (F)** Flow cytometry was used to measure JC-1 of OS cells. **(G)** Effect of ERKi on GPx and GSH levels in OS cells. **(H)** Immunofluorescence co-staining of mitochondria in OS cells 24 h post-ERKi treatment **(I)** Cell-survival rate in different groups (*n* = 5 per group; **p < 0.01 versus NC group; *p < 0.05 versus NC group).

**FIGURE 4:**
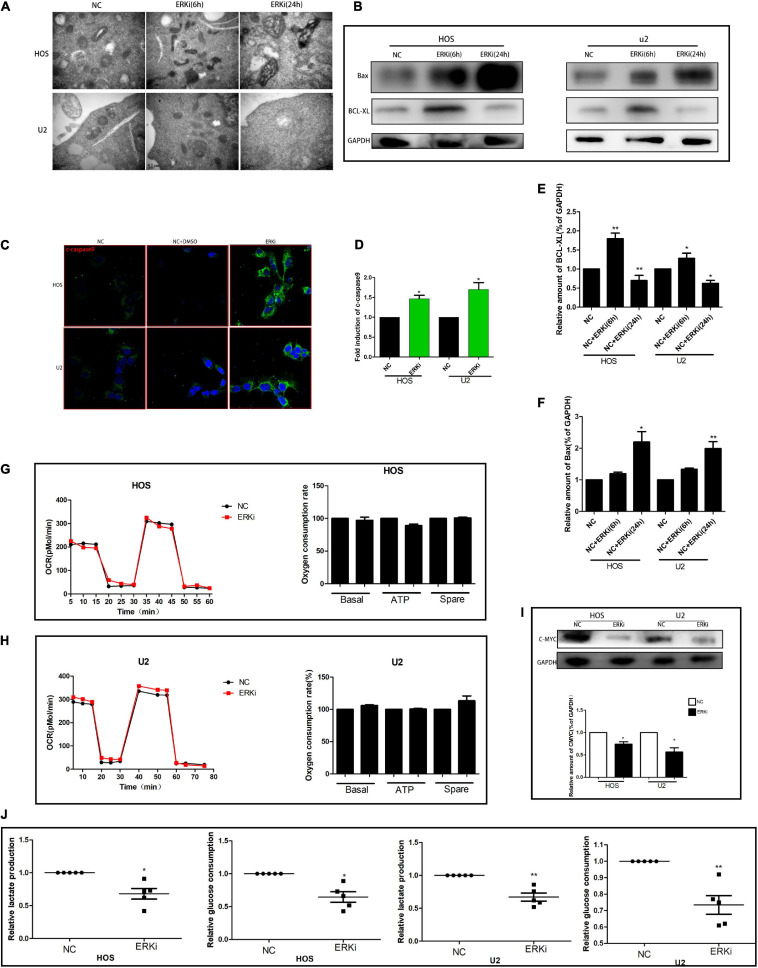
ERK inhibition promotes apoptosis and regulates aerobic glycolysis in OS cells. **(A)** TEM images of ERKi-treated OS cells after 6 h and 24 h of treatment (black arrows). **(B)** Protein expression of Bax and BCL-XL after treatment with ERKi; GAPDH was used as the loading control and for band density normalization. **(C,D)** Immunofluorescence staining of c-caspase9 after 24 h of treatment with ERKi. **(E,F)** Optical density of Bax and BCL-XL proteins. **(G,H)** Diagram of OCR results obtained by OROBOROS Oxygraph-2k. **(I)** Western blotting and densitometric quantification. **(J)** Glucose uptake and production of lactic acid in OS cell lines in different groups (*n* = 5 per group; **p < 0.01 versus NC group; *p < 0.05 versus NC group).

The level of ROS in OS cells increased evidently when the cells were exposed to ERKi for an extended duration (Figures 3A,B). As shown in Figures 3C–F, at 24 h post-treatment, NC cells stained with JC-1 emitted red fluorescence and some green fluorescence; compared to this group, the ERKi-treated group produced more green fluorescence, showing an increased green/red fluorescence ratio. ERKi also suppressed the amount of intracellular GSH and the activity of glutathione peroxidase (GPX) in OS cells (Figure 3G), and GSH precursor antioxidant NAC and mitochondrial membrane PT pore inhibitor cyclosporine A (CsA, 5 μM) pre-treatment in OS cells significantly reversed ERKi-induced cell death (Figure 3I). Compared with the control group, the treatment group showed spotty fluorescent aggregation of the mitochondria (Figure 3H). Microstructural changes in the mitochondria of OS cells observed using TEM showed that in the early stages of ERKi treatment, numerous mitochondria were present that were small in size. With an extension in treatment with ERKi, the number of mitochondria decreased, edema occurred, and mitochondrial ridges became blurred (Figure 4A). The expression levels of Bax (Figures 4B,F) and active-caspase9 protein (Figures 4C,D) were significantly upregulated during ERKi treatment, and the expression of BCL-XL protein showed the opposite trend (Figures 4B,E); in contrast, no such difference was observed in the early stage of OS cell treatment with ERKi (Figures 4B,E). Our data also showed that ERKi suppressed the aerobic glycolytic capacity (Figure 4J) and induced a decrease in c-myc protein level in OS cells (Figure 4I). Besides, ERKi does not affect the oxygen consumption of OS cells in the early stages as reflected by OCR measurement (Figures 4G,H). In summary, ERKi induced mitochondrial apoptosis and abnormal glycolysis in OS cells. In the early stage, OS cells demonstrate an energy intake disorder. Thus, there were compensatory increases in mitochondrial activity, but no productivity was found.

### Effect of HCQ on OS Cells

**FIGURE 5:**
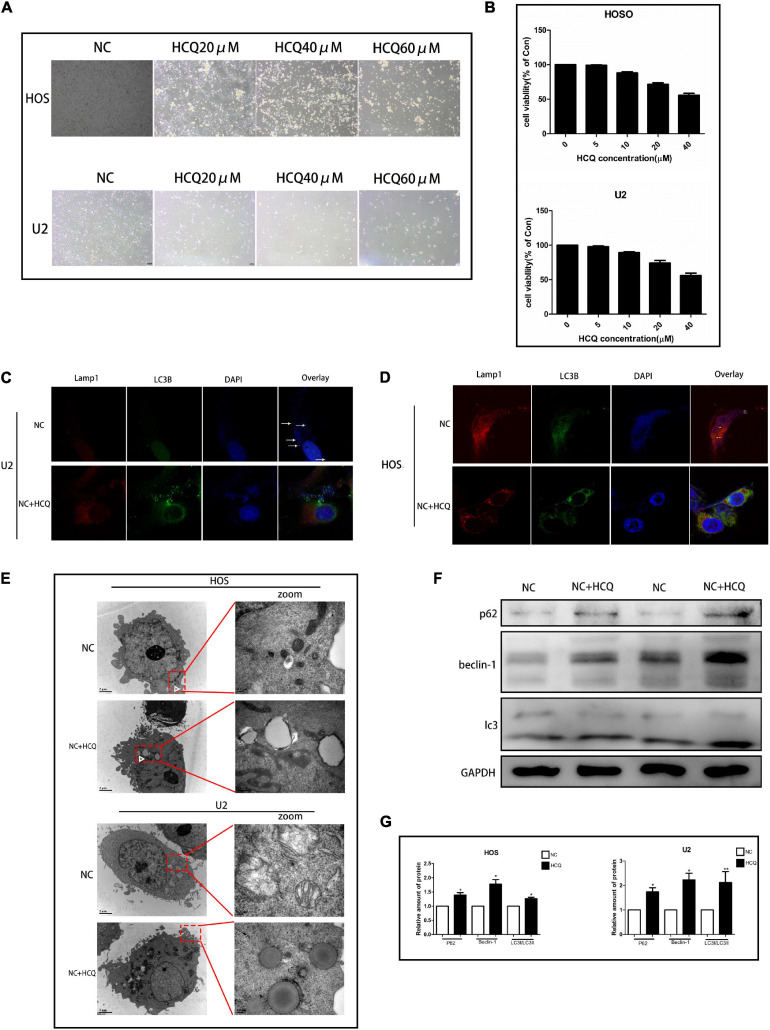
HCQ inhibits the activity of OS cells. **(A)** Cell morphology. **(B)** CCK8 proliferation analysis. **(C,D)** Immunofluorescence co-staining of Lamp-1 (red) and LC3B (green) in OS cells 24 h post-HCQ treatment. **(E)** TEM images of HCQ-treated OS cells after 6 h and 24 h. Autophagosomes are indicated by white arrows. **(F,G)** Western blotting and densitometric quantification (*n* = 5 per group; **p < 0.01 versus NC group; *p < 0.05 versus NC group).

To explore the effect of HCQ on OS cells, we examined cellular morphology and survival. The survival rate of OS cells treated with HCQ gradually decreased in a concentration-dependent manner (Figure 5B). The morphological changes in cells were consistent with the results of survival rate. With an increase in HCQ concentration, the morphology of OS cells shrank and became deformed (Figure 5A). The morphology of OS cells was observed using TEM. The number of autophagosomes was significantly increased after treatment with HCQ compared with the number in the NC group (Figure 5E). Simultaneously, we also found that the number of lysosomes did not decrease. Moreover, we performed immunofluorescence co-staining analysis of autophagy-related proteins LC3B and Lamp-1 in OS cells. Our findings indicated that co-localization of LC3B and Lamp-1 proteins was significantly reduced in the HCQ-treated OS cells compared with the untreated cells (Figures 5C,D). Furthermore, HCQ treatment increased the expression of LC3B and Beclin-1 proteins (Figures 5F,G) in early autophagy.

### Combination of ERKi and HCQ Synergistic Inhibition of OS Cell Activity

**FIGURE 6:**
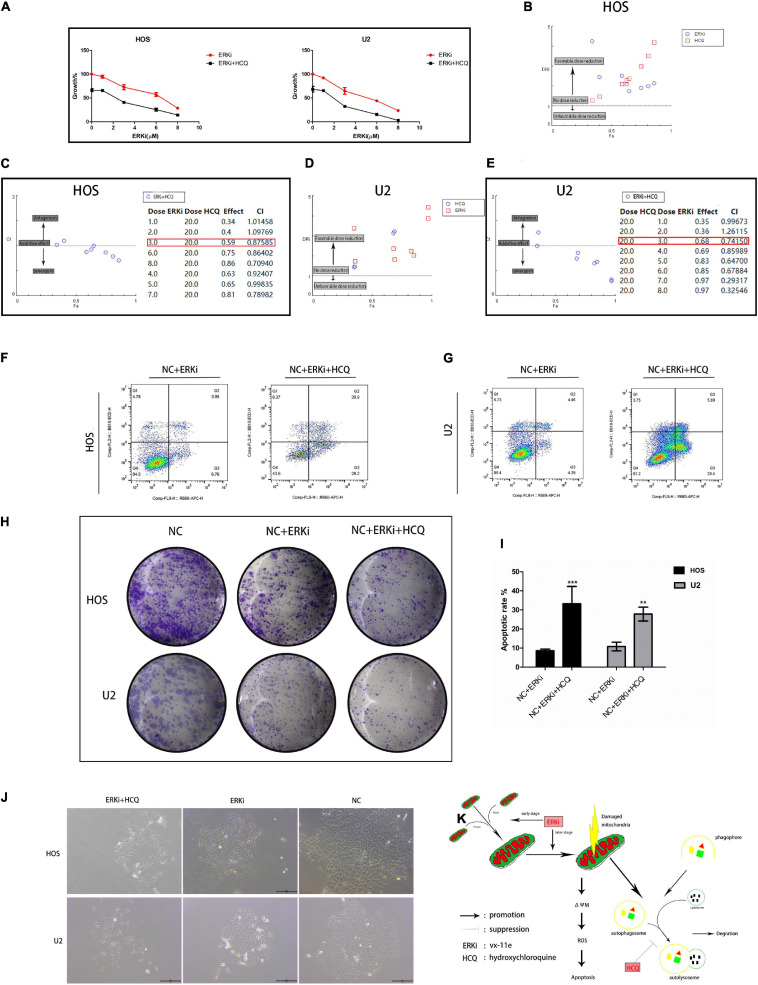
Synergistic inhibitory effect of ERKi and HCQ combined treatment on OS cell activity. (**A**) OS cells were treated with ERKi and HCQ (20 μM) for 24 h. Proliferation was assessed using live-cell counting. (**B,D**) Dose reduction index (DRI) for each drug for a given effect (Fa). DRI of > 1, 1, and < 1 indicate a favorable dose reduction, no dose reduction, and a negative dose reduction, respectively. **(C,E)** Combination index (CI) values as a function of the effect levels (Fa), where CI values of > 1, 1, and < 1 indicate synergism, an additive effect, and antagonism, respectively. **(F,G,I)** Flow cytometry to determine the percentage of total apoptotic OS cells. **(H,J)** Colony-forming assay for OS cells. **(K)** Schematic representation of the synergistic effects of ERKi and HCQ on OS cells (n = 5 per group; ***p < 0.001 versus NC+ERKi group; **p < 0.01 versus NC group).

To verify the synergistic inhibition of OS cell activity by HCQ and ERKi, the growth, proliferation, migration, and apoptosis of OS cells were investigated. In CCK8 analysis, compared with OS cells treated with ERKi alone, the combined use of HCQ had a more pronounced inhibitory effect on OS cell growth (Figure 6A). The synergism or antagonism of the drug combination of ERKi and HCQ in OS was analyzed using CompuSyn software. The combination of ERKi and HCQ in OS indicated a favorable dose reduction (Figures 6B,D) and played a synergistic role (Figures 6C,E). To further characterize the highly tumorigenic capacity of OS cells, we conducted a colony-formation assay. A large and well-spread colony had formed in the NC group. Further, ERKi restrained colony formation, but the combination of ERKi and HCQ had a more potent antitumor effect than ERKi alone (Figure 6J). Furthermore, proliferation results indicated that the combination of ERKi and HCQ had more inhibitory effects than ERKi alone on OS cell proliferation (Figure 6H). Cell apoptosis was detected using flow cytometry after annexin V-APC/7-AAD (MultiSciences, China) double staining. Both the percentage of early apoptotic properties and the total apoptotic properties were higher in the group with ERKi treatment than in the NC group; the combined HCQ and ERKi treatment showed an accelerated apoptotic effect (Figures 6F,G,I).

### Combination of ERKi and HCQ Synergistic Inhibition of OS *in vivo*

**FIGURE 7:**
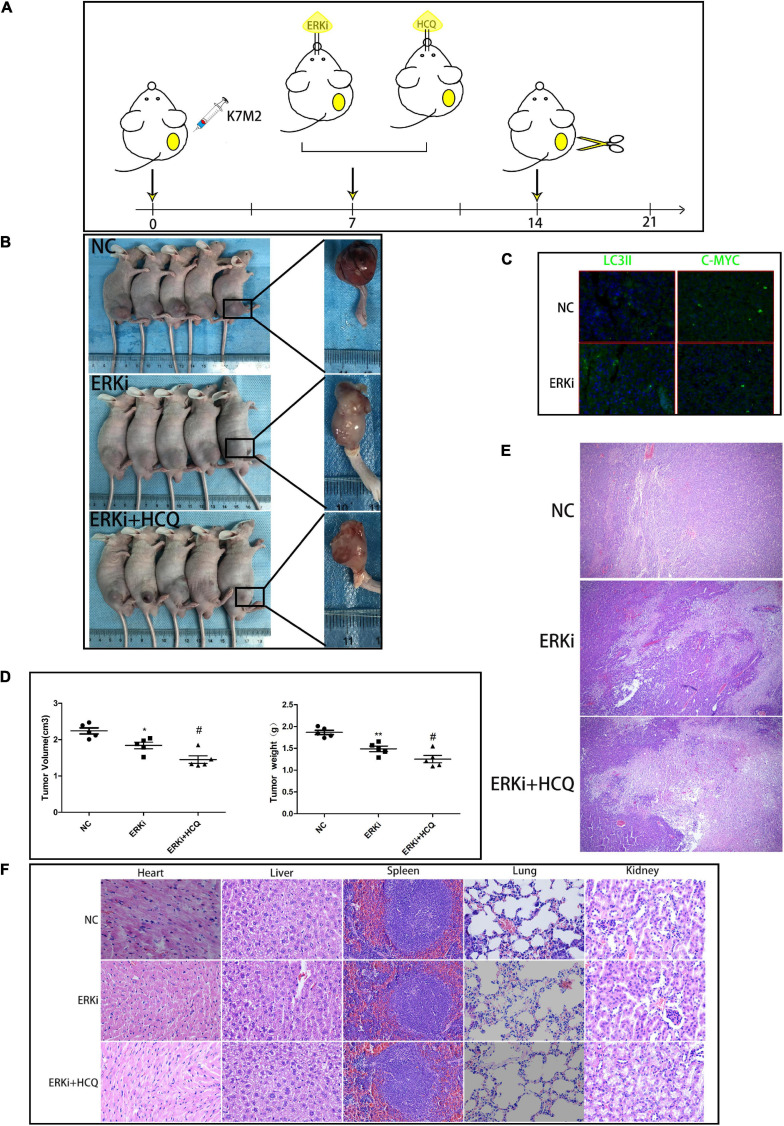
Synergistic inhibitory effect of ERKi and HCQ combined treatment on OS *in vivo*. **(A)** Schematic representation of treatment regimen and surgery. **(B,D)** Tumor formation in different groups. **(C)** Immunofluorescence staining to determine the levels of LC3B and CMYC. **(E)** H&E staining of OS tissue among groups. **(F)** H&E staining of organ tissues of mice administered ERKi or HCQ (*n* = 5 per group; **p < 0.01 versus NC group; *p < 0.05 versus NC group; ^#^*p* < 0.05 versus ERKi group).

We used a right tibial tumor model in mice and performed the treatment process shown in Figure 7A. The physical photos of these excised tibial tumors and corresponding volume and weight charts (Figures 7B,D) indicated that there was a difference in tumor sizes and weights among the three groups; the data showed that the combination of ERKi and HCQ significantly inhibited tumor growth *in vivo*. H&E staining revealed more tumor cell death in the ERKi + HCQ group (Figure 7E) compared to the other groups. Immunohistochemical quantitative analysis confirmed that the expression level of LC3B was higher in the ERKi group than in the NC group, indicating that ERKi promoted autophagic flux of OS *in vivo*. Furthermore, the expression levels of C-MYC were downregulated in the ERKi group compared to the NC group (Figure 7C). Moreover, as shown in Figure 7F, H&E staining indicated that no histological damage had been observed in multiple organs in the ERKi group and ERKi + HCQ group.

## Discussion

ERK is a key downstream protein in the RAS-RAF-MEK-ERK signaling pathway, and its overactivation has been demonstrated in various tumors (Asati et al., 2016). At present, antitumor drugs targeting this pathway, including BRAF inhibitors (vemurafenib, sorafenib, and dabrafenib) as well as MEK inhibitors (such as selumetinib, trametinib, and cobimetinib), have been marketed and have shown to be efficacious in clinical trials. However, it is inevitable that the tumor cells will acquire drug resistance during treatment with this kind of drug. ERK signaling pathways are associated with a variety of substrates such as kinases and transcription factors, and once drug-resistant mutations occur in cells, it causes cell death. Thus, acquired mutations of ERK1/2 are almost absent in tumor cells. Therefore, ERK-targeting drugs are undoubtedly a promising anticancer agent. Previous studies have shown that innate resistance to MEK or RAF inhibitors was reactivated through ERK1/2 in tumor cells (Kidger et al., 2018). Based on the abovementioned studies, compared with RAF inhibitors and MEK inhibitors, ERKis have antitumor effects and can prevent tumor cells from developing drug resistance to a certain extent; thus, they have a broader application prospect in clinical practice. Although no ERK1/2 inhibitor has been officially approved for use, a number of small molecules of ERKis, including Ulixertinib (Sullivan et al., 2018), KO-947, and MK-8353 (Moschos et al., 2018), are currently in the preclinical development phase.

The relationship between autophagy and tumor is complicated: on the one hand, in human tumors, autophagy genes are frequently silenced or mutated, leading to increased stress and tumor progression and indicating that autophagy plays a tumor-suppressor role (Kung et al., 2011; Mowers et al., 2018); however, on the other hand, given the characteristics of abnormal proliferation of tumor cells, their environment has a higher degree of metabolism and other pressures than normal cell environment, making them more dependent on autophagy for their survival (Srinivasan et al., 2017). Furthermore, research on the regulation of autophagy to combat antitumor drug resistance is expanding and becoming increasingly important. TFEB, as a regulatory tool for lysosomal biogenesis, plays an important role in autophagy. TFEB phosphorylation forms a 14–3–3 docking target, resulting in a deactivated TFEB trapped in the cytoplasm (Xu et al., 2019). When TFEB is activated for nuclear transfer, it transmits information about the lysosomal state to the nucleus to trigger a transcriptional response, thereby stimulating autophagy. Further, TFEB nuclear translocation, in association with MAPK/ERK, has been confirmed in a steatohepatitis model (Kim et al., 2017). We found that acute ERK inhibition increases rather than decreases the autophagic flux in OS cells. We believed that this phenomenon may have occurred because acute ERK inhibition regulates TFEB nuclear translocation in OS (Figure 2J). As expected, ERKi exacerbated TFEB nuclear translocation in OS cells (Figures 1H,I). The molecular mechanisms underlying autophagy inhibition were extremely complex and involved multiple molecules. To further explore this specific mechanism, we used HCQ, an FDA-approved late-stage autophagy inhibitor used in clinical trials, to block the fusion of autophagosomes with lysosomes to inhibit the late stage of autophagy. As expected, HCQ exacerbated the death of ERKi-treated OS cells. In other words, autophagy was activated by ERKi as a compensatory response to maintain cell homeostasis, which in turn further enhanced the dependence of OS cells on autophagy.

Although tumor cells prefer glycolysis as the main mode of energy production and are more dependent on autophagy to remove metabolic waste (Levy et al., 2017), mitochondria still play an important role in the occurrence and development of tumors. In response to the presence of abundant oxygen, the mitochondrial phosphoric acid oxidation is still active. Furthermore, some enzymes needed for glycolysis are located in the mitochondria (Porporato et al., 2018). Moreover, mutations, alterations, and deletions in mitochondrial DNA have been reported in many cancers (Nikolaev, 2018). Recent studies suggest that mitochondrial dynamics and dysfunction are the links among mitochondrial DNA defects, excessive fission, mitochondrial retrograde signaling, and cancer progression (Srinivasan et al., 2017). Mitochondrial oxidative phosphorylation is not impaired in tumor cells and plays an important role in the malignant progression of tumors. A previous study pointed out that mitochondrial dysfunction and oxidative stress trigger a niche favoring cholangiocellular overgrowth and tumorigenesis (Yuan et al., 2017). Moreover, the regulation of ROS plays a very important role in the treatment of cancer. Therefore, some chemoradiotherapy drugs interfere with the outcome of tumors by regulating ROS (Sosaa et al., 2013). Our data showed that ERKi treatment inhibited the activity of OS by increasing the expression of ROS and damaging the antioxidant system. Additionally, ERK induces mitochondrial translocation of phosphoglycerate kinase1 (PGK1) to regulate cancer metabolism and tumorigenesis (Li et al., 2016). Our results showed that in the early stages of ERKi treatment in OS cells, the mitochondria showed a compensatory state, including the increased number and increased expression of fission factors. Later, mitochondrial apoptosis seems to have occurred. This may be the case in the ERKi-treated OS cells: glycolysis activity was suppressed, causing an intake energy disorder in OS cells; thus, there were compensatory increases in mitochondrial activity (Figure 6K). However, such compensation is impossible following ERKi-treatment, which may induce mitochondrial apoptotic-related gene expression in OS cells.

## Conclusion

In this study, we found that inhibition of ERK signaling via pharmacological methods could lead to enhanced anti-OS activity of the autophagy inhibitor. Mechanistically, ERK inhibition directly regulated the nuclear translocation of TFEB to increase autophagy flux and promote mitochondrial apoptosis through the ROS/mitochondria pathway and indirectly increased the dependence of OS cells on autophagy. In addition, we found changes in the mitochondrial dynamics in OS cells during the early stages of ERKi treatment. This may be a manifestation of mitochondrial compensatory tumor promotion, caused by the restraining of aerobic glycolysis by ERKi and the subsequent OS energy-intake disorder.

## Data Availability Statement

The raw data supporting the conclusions of this article will be made available by the authors, without undue reservation.

## Ethics Statement

The animal study was reviewed and approved by the Research Ethics Committee of Zhejiang University.

## Author Contributions

YQ conceived and designed the research. MZ and YB performed the experiments. CX and YQ analyzed the data and interpreted results of the experiments. JM, WZ, and HS drafted and reviewed the manuscript. All authors contributed to the article and approved the submitted version.

## Conflict of Interest

The authors declare that the research was conducted in the absence of any commercial or financial relationships that could be construed as a potential conflict of interest.

## Publisher’s Note

All claims expressed in this article are solely those of the authors and do not necessarily represent those of their affiliated organizations, or those of the publisher, the editors and the reviewers. Any product that may be evaluated in this article, or claim that may be made by its manufacturer, is not guaranteed or endorsed by the publisher.

## Footnotes

1
http://www.cellbank.org.cn
